# Integrated Surveillance of *Trichinella* spp. and Rabies Virus-Neutralising Antibodies in Golden Jackals (*Canis aureus*) and Red Foxes (*Vulpes vulpes*) from Western Romania

**DOI:** 10.3390/ani16081135

**Published:** 2026-04-08

**Authors:** Maria Roberta Tripon, Cristina Mirabela Gaspar, Răzvan Tudor Pătrînjan, Renata Knop, Răducu Cristian Marinaș, Florinel Cosmin Boja, Florin Adrian Huiban, Claudia Daniela Șerban, Camelia Tulcan

**Affiliations:** 1 Faculty of Engineering and Applied Technologies, University of Life Sciences “King Mihai I” from Timișoara, 119 Calea Aradului, 300645 Timișoara, Romania; roberta.tripon@usvt.ro (M.R.T.); cristian.marinas@usvt.ro (R.C.M.); florinelcosmin.boja@usvt.ro (F.C.B.); cameliatulcan@usvt.ro (C.T.); 2 Doctoral School of Plant and Animal Resources Engineering, University of Life Sciences “King Mihai I” from Timișoara, 119 Calea Aradului, 300645 Timișoara, Romania; florin.huiban@usvt.ro (F.A.H.); claudia.nastase.iosud@usvt.ro (C.D.Ș.); 3 Faculty of Veterinary Medicine, University of Life Sciences “King Mihai I” from Timișoara, 119 Calea Aradui, 300645 Timișoara, Romania; cristina.gaspar@usvt.ro; 4 Doctoral School of Veterinary Medicine, University of Life Sciences “King Mihai I” from Timisoara, 119 Calea Aradui, 300645 Timișoara, Romania; 5 Department of Animal Science, Institute of Animal Science, Biotechnology and Nature Conservation, Faculty of Agricultural and Food Sciences and Environmental Management, University of Debrecen, 4032 Debrecen, Hungary

**Keywords:** *Trichinella* spp., rabies, *Canis aureus*, *Vulpes vulpes*, wildlife reservoirs, oral rabies vaccination, zoonotic risk, Romania, disease surveillance, One Health

## Abstract

The rapid expansion of the golden jackal (*Canis aureus*) across Eastern Europe has reshaped mesocarnivore communities, with potential influence on the dynamics of zoonotic disease. In Romania, where both *Trichinella* spp. and rabies remain public health concerns, updated data on wildlife reservoirs are essential. This study aims to provide an integrated assessment of *Trichinella* prevalence and rabies virus-neutralising antibody (RVNA) profiles in 134 wild canids (96 golden jackals and 38 red foxes, *Vulpes vulpes*) from northwestern Romania (August 2025–January 2026). *Trichinella* larvae were detected using the artificial digestion method, and infection intensity was expressed as larvae per gram. Rabies serology was performed using a commercial ELISA kit, with 0.5 IU/mL considered the protective threshold. *Trichinella* prevalence was significantly higher in foxes (78.9%) in comparison with jackals (60.4%), with similar larval burdens in both species. More than half of the individuals in both species exhibited RVNA titers below the protective threshold, indicating heterogeneous immunity levels in the population. No significant age- or sex-related differences in seroconversion were observed. These findings confirm intense sylvatic circulation of *Trichinella* spp. and highlight potential immunity gaps in wildlife rabies control. The results support the need for integrated, multi-pathogen surveillance and explicit inclusion of the golden jackal in disease monitoring and management strategies.

## Simple Summary

In Romania, certain wildlife-borne diseases, such as trichinellosis (a parasitic infection transmitted through raw or undercooked meat) and rabies (a fatal viral disease affecting the nervous system), remain important risks for both animals and people. The rapid expansion of the golden jackal is raising questions about its role in disease maintenance and transmission, alongside the red fox. This study examined 96 jackals and 38 foxes from western Romania to determine both the occurrence of *Trichinella* infection and the presence of rabies virus-neutralising antibodies (RVNA). We found a high rate of *Trichinella* infection in both species, especially in foxes, and more than half of the animals had insufficient protective antibody levels against rabies, suggesting possible gaps in population immunity. These findings indicate that both jackals and foxes may play an important role in maintaining disease in wildlife. Monitoring these species more closely would help improve rabies control programs and reduce the risk of parasite transmission to domestic animals and humans, supporting public health and responsible wildlife management.

## 1. Introduction

The Eurasian golden jackal (*Canis aureus*) (Linnaeus, 1758) is a medium-sized canid, characterised by ecological plasticity and omnivorous feeding habits. Native to parts of Africa and Asia, it has undergone a remarkable range and number expansion across Europe over the past few decades [1]. Originally documented in southeastern Europe, this highly adaptable species has progressively colonized new territories westward and northward, reaching Central Europe [2] and the Baltic region [3].

Across Romania, a country located at the ecological intersection of the Balkans, the Carpathians, and the Danube Delta, the golden jackal has experienced a rapid demographic increase and westward expansion, rising from roughly 1291 to over 40,000 individuals between 2004 and 2025 and shifting from an earlier concentration in the southern and southeastern regions to a near-complete colonization of lowland and hilly areas nationwide, with the highest densities in southern Romania (including the Danube Delta, Dobrogea, and Oltenia) and notable clusters in western Banat, southern and eastern Muntenia, and parts of Moldova, while only mountainous or heavily forested regions remain sparsely occupied [4]. This trend is consistent with broader continental expansion patterns observed throughout Europe and is attributed to climate warming, landscape fragmentation, increased availability of anthropogenic food sources, and reduced competition from apex predators [5,6,7].

This expansion has led to a high nutritional niche overlap with the red fox (*Vulpes vulpes*) (Linnaeus, 1758), a small-sized, highly adaptable and opportunistic carnivore. In Romania, the red fox is the main species causing damage to small game, occasionally large game, as well as domestic species, especially poultry [8]. Evidence indicates that, rather than causing a sharp decline in red fox populations, the presence of golden jackal in Romania has primarily driven behavioural (avoidance) and physical adaptations of the foxes [9,10]. In fact, some studies show the red fox population itself saw a major increase, from 40,956 to 78,784 individuals between 1999 and 2023 [8]. To maintain sympatry with the larger and competitively dominant jackal, red foxes appear to adjust their spatial behaviour, particularly by exploiting peri-urban and rural habitats where human-derived food resources buffer them from competitive pressures and help sustain stable or even increasing densities [11,12].

Golden jackal and red fox populations, when healthy, ecologically balanced, and properly size-controlled, can provide important ecological benefits. As adaptable mesopredators, they help regulate rodent populations, protecting crops and maintaining trophic balance. Their scavenging behaviour removes carrion and organic waste, contributing to environmental sanitation and potentially reducing sources of infection. Red foxes also support plant regeneration by dispersing seeds through their faeces, enhancing vegetation diversity [13,14].

However, their adaptability and mobility also generate ecological and health concerns. From both ecological and epidemiological perspectives, they function as highly adaptable “bridge species,” connecting wild ecosystems with human-dominated environments [15]. Their ability to exploit anthropogenic food sources such as garbage increases their presence near human settlements, raising the likelihood of contact with pets, livestock, and people. Such interactions may lead to livestock predation, poultry losses, or occasional aggressive encounters. In areas where they are in high density, they can become invasive, threatening local bird and small mammal populations [4,8,13,14].

Epidemiologically, both species can act as reservoirs and sentinels for zoonotic pathogens, including rabies, trichinellosis, echinococcosis, and other parasitic, bacterial or viral infections, posing risks to domestic animals and human health [15,16,17]. Among pathogens of major significance in Romania, *Trichinella* spp. and the rabies virus remain priority concerns due to their persistence in wildlife and their importance to public health [18,19].

Romania hosts one of the most active *Trichinella* sylvatic cycles in Europe, with high prevalence in carnivores such as foxes, jackals, wolves, and wildcats [20,21]. *Trichinella britovi* (Pozio, La Rosa, Murrell, and Lichtenfels, 1992) predominates, while *Trichinella spiralis* (Owen, 1835) and the emerging *Trichinella pseudospiralis* (Garkavi, 1972) have also been identified in wild carnivores, including recent detections in Romanian jackals [22,23]. The scavenging ecology of red fox and golden jackal makes them highly competent maintenance hosts, facilitating continuous parasite circulation and spillover risk to domestic pigs and humans [24].

Similarly, rabies, a fatal viral disease caused by rabies lyssavirus belonging to the *Rhabdoviridae* family, remains a persistent threat despite the implementation of nationwide oral rabies vaccination (ORV) campaigns in wildlife since 2012. Interruptions or inconsistencies in ORV delivery (2018, 2021, and 2022) have coincided with renewed rabies outbreaks in northern and eastern Romania, largely concentrated in border areas with Ukraine, including 28 confirmed animal outbreaks and a fatal human case reported in July 2025 [25,26]. Red foxes are the primary rabies reservoir in Europe, but the expanding golden jackal population, with its mobility and synanthropic behaviour, may also influence rabies dynamics [27]. Serological monitoring through rabies virus-neutralising antibody (VNA) detection is essential for assessing ORV effectiveness and exposure in wildlife populations [28].

Despite Romania’s long-standing challenges with trichinellosis and rabies, current epidemiological data on these pathogens in wild carnivores, particularly in the context of recent golden jackal expansion, remain limited. There is a strong need for an improved risk evaluation, targeted surveillance, and evidence-based wildlife disease management in our country. Thus, the present study aimed to address these knowledge gaps by providing an integrated assessment of *Trichinella* prevalence and rabies VNA profiles in two ecologically influential carnivore species—golden jackal and red fox.

## 2. Materials and Methods

### Ethical Considerations

The animals included in this study were legally harvested during routine hunting activities and not killed specifically for research purposes. As golden jackals and red foxes may be hunted year-round in Romania under national wildlife management regulations [29,30], and sampling was conducted post-mortem, the study was exempt from Animal Research Ethics Committee review at the University of Life Sciences “Regele Mihai I” from Timișoara, Romania.

**Figure 1:**
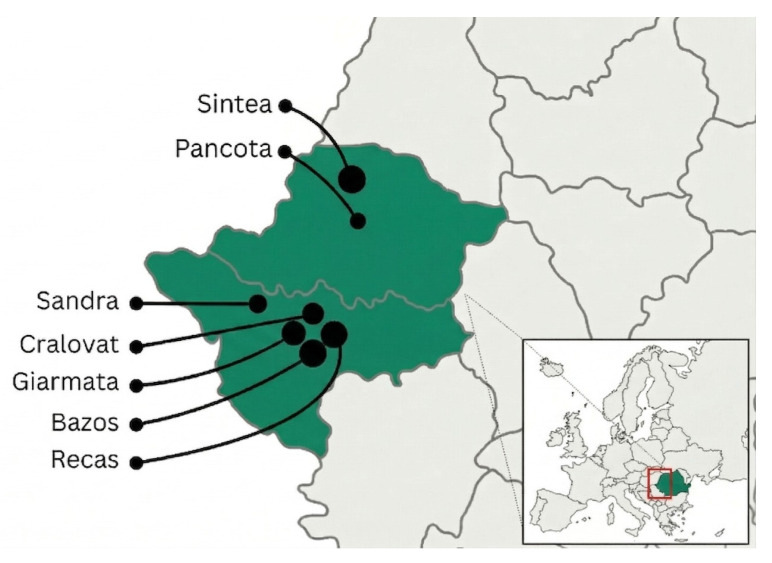
Study area.

The study was conducted in seven hunting grounds located in western Romania: Sintea (Arad County), Pancota (Arad County), Șandra (Timiș County), Giarmata (Timiș County), Bazoș (Timiș County), Recaș (Timiș County) and Cralovăț (Timiș County) (Figure 1). These areas are characterized by a mosaic of agricultural land, peri-urban and rural habitats, forest patches and wetlands.

Samples were collected during legal hunting expeditions between August 2025 and January 2026, from a total of 134 wild carnivore carcasses. The sampled population consisted of 38 hunted individuals of red fox and 96 of golden jackal. Both species can be legally hunted year-round in Romania. Animals included in the study were harvested under the annual quota approved by the Minister of the Environment, Water and Forests [29,30]. For each animal, muscular tissue (*n* = 134) and blood samples (*n* = 134) were collected to assess the presence of *Trichinella* spp. larvae, and for serological detection of rabies virus-neutralising antibodies (RVNA), respectively. Among the 38 foxes, the sex and age distribution was as follows: 22 males, 11 females and 5 juveniles (4 females, 1 male). Among the 96 jackals, the distribution was: 55 males, 14 females and 27 juveniles (13 females, 14 males).

The biological samples were collected in the field immediately after shooting and confirmation of death. For each individual, blood samples were collected into clot-activator tubes for serum separation by puncture of the thoracic or abdominal aorta. Muscle samples consisted of diaphragmatic pillars. During transport, samples were stored in portable coolers at 2–8 °C. In the laboratory, blood samples were refrigerated at 2–8 °C, serum was separated and stored at −80 °C until analysis, and muscle tissue was refrigerated at 2–8 °C and processed within 24 h after harvesting.

The detection of *Trichinella* spp. larvae were performed using the artificial digestion method in accordance with Commission Implementing Regulation (EU) 2015/1375 [31]. Approximately 5 g of striated muscle tissue per animal (diaphragmatic pillars), trimmed of fat and connective tissue, were subjected to enzymatic digestion. Samples were incubated in 2.0 L of water preheated to 46–48 °C, containing 10 g of pepsin (1:10,000 NF) and 16 mL of 25% hydrochloric acid. Digestion was carried out for 50 min, under constant magnetic stirring until complete tissue dissolution. The digest was subsequently filtered and allowed to sediment. The sediment was examined microscopically for the presence of *Trichinella* larvae. Larval counts were expressed as larvae per gram (LPG) of muscle tissue. This method is considered the reference technique for wildlife surveillance due to its high sensitivity.

Serological detection of rabies virus-neutralising antibodies was performed using the Rabies Virus Antibodies ELISA Kit (E-AD-E069, Elabscience, Wuhan, China, 96 T format). The kit is based on an indirect enzyme-linked immunosorbent assay (ELISA). Microtiter plates are pre-coated with recombinant rabies virus (RBV) antigen. Serum antibodies specific to rabies virus bind to the coated antigen, followed by the addition of horseradish peroxidase (HRP) conjugate. After substrate addition, color development is proportional to antibody concentration. Optical density (OD) was measured at 450 nm (reference 630 nm).

Serum samples were diluted 1:100 in sample diluent before testing (2 µL serum + 198 µL diluent). Standards were prepared from the Rabies Positive Serum National Standard (CVCC Z53) to generate a 4 IU/mL stock solution, followed by serial dilutions to produce a standard curve (4, 2, 1, 0.5, 0.25, 0.125, and 0 IU/mL). All samples and standards were tested in duplicate.

Antibody concentrations (IU/mL) were calculated using a four-parameter logistic standard curve. Results were interpreted according to the manufacturer’s criteria: <0.125 IU/mL—undetectable seroconversion; 0.125–0.5 IU/mL—insufficient seroconversion; 0.5–4 IU/mL—sufficient seroconversion; >4 IU/mL—high seroconversion level. For epidemiological analysis, titers were also categorised as non-protective (<0.5 IU/mL) and protective (≥0.5 IU/mL).

Prevalence was calculated as the proportion of positive individuals among the total examined, with 95% confidence intervals (CI). Differences in prevalence between species, sex and age categories were assessed using Pearson’s chi-square test. Larval burden data (LPG) and antibody titers were tested for interspecific and age/sex-related differences using Mann–Whitney U tests. Statistical significance was set at *p* < 0.05. Data analyses were performed using the online statistical software Statistics Kingdom [32].

## 3. Results

### 3.1. Prevalence of Trichinella spp.

**Figure 2:**
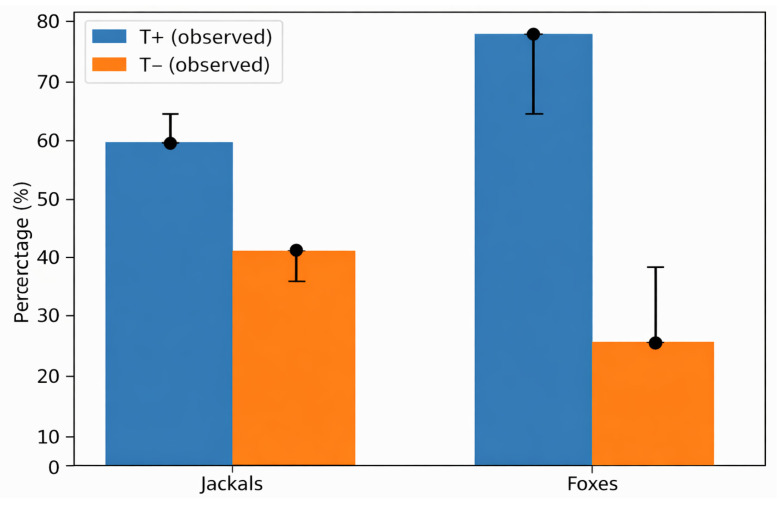
Prevalence of *Trichinella* spp. infection by species—observed frequencies (T+ positive, T− negative), with expected frequency represented as unidirectional error bars.

The prevalence of *Trichinella* infection in golden jackals was 60.4% (58/96; 95% CI: 50.4–69.6%) and 78.9% in red foxes (30/38; 95% CI: 63.7–89.3%), with a statistically significant difference between the two species (χ^2^ = 4.14, *p* = 0.041) (Figure 2).

**Figure 3:**
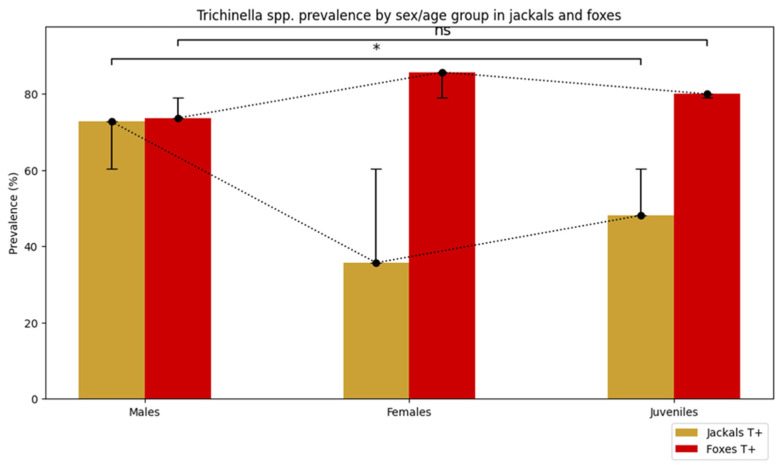
Observed *Trichinella* prevalence (T+) in golden jackals and red foxes across sex and age categories (males, females, juveniles). Bars show observed frequencies, with unidirectional error bars representing the expected frequencies. Jackals display significant differences among categories (*), while foxes show no significant variation (ns).

Among jackals, *Trichinella* infection varied significantly across biological categories (males, females, juveniles) (χ^2^ = 8.76, *p* = 0.012). Infection prevalence was highest in males, with 72.7% testing positive (40/55; 95% CI: 59.8–82.7%), followed by juveniles at 48.1% (13/27; 95% CI: 30.7–66.0%), and lowest in females at 35.7% (5/14; 95% CI: 16.3–61.2%) (Figure 3).

Larval densities ranged from 0.2 to 78.8 larvae per gram (LPG), with an overall mean intensity of 15.5 ± 13.9 LPG and a median of 15 LPG. Adult males (*n* = 40) showed infection intensities between 4.4 and 52.6 LPG, with a mean of 18.45 ± 9.1 LPG and a median of 15.9 LPG. Adult females (*n* = 5) exhibited a wider dispersion of values, ranging 4.0–40.2 LPG (mean 14.7 ± 14.7 LPG, median 10.4 LPG). Juveniles (*n* = 13) demonstrated generally lower infection intensities, ranging 0.2 to 78.8 LPG, with a mean of 6.9 LPG, median of 1.0 LPG, and a high standard deviation of 21.6 driven by a single heavily infected juvenile (78.8 LPG). When comparing adult males and females in terms of larval burden, there were no statistically significant differences (U = 149.0, *p* = 0.079). The small number of adult females limits the statistical power of this comparison.

In red foxes, *Trichinella* infection prevalence was high across all biological categories, without statistically significant differences (*p* = 0.855, Freeman-Halton extension of the Fisher exact probability test) (Figure 3). Males exhibited a prevalence of 73.7% (14/19; 95% CI: 51.2–88.2%), females 85.7% (12/14; 95% CI: 60.1–96.0%), and juveniles 80.0% (4/5; 95% CI: 37.6–96.4%).

Infection intensities in red foxes varied widely, ranging from 0.4 to 55.4 LPG, with an overall mean of 13.1 ± 12.9 LPG and a median of 9.9 LPG. Adult males (*n* = 14) showed the highest infection intensities, ranging from 3.0 to 40.4 LPG, with a mean of 14.4 ± 10.9 LPG and a median of 10.7 LPG. Adult females (*n* = 12) displayed a broader range (1.6–55.4 LPG) but a lower central tendency (mean 12.6 ± 14.6 LPG, median 8.9 LPG). Juveniles (*n* = 4, all females) generally exhibited lighter but more variable infection levels, ranging from 0.4 to 36.4 LPG, with a mean of 10.1 ± 17.6 LPG and a median of 1.8 LPG. The highest value recorded in the fox population was 55.4 LPG, observed in an adult female. Surprisingly, another high value of 36.4 LPG was recorded for a juvenile. There were no statistically significant differences in terms of infection intensities between adult males and females (U = 66, *p* = 0.367).

**Figure 4:**
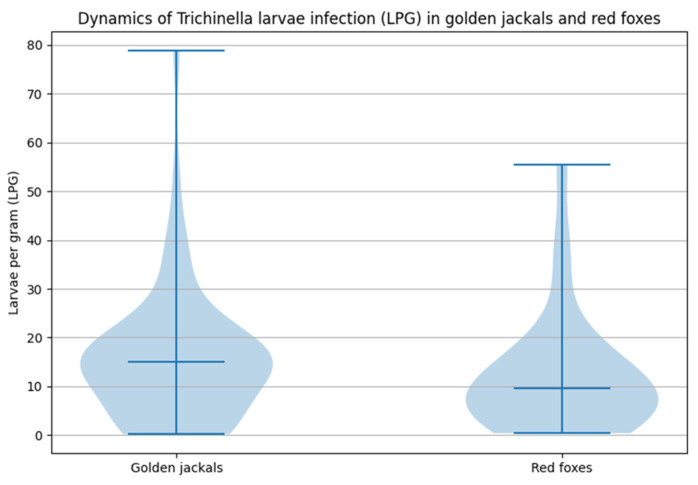
The distribution of infection intensities in golden jackals (*n* = 58) and red foxes (*n* = 30). Jackals exhibit a wider and more variable range of LPG values, including several heavily infected individuals, while foxes display a more compact distribution. Median values are indicated by horizontal lines.

The difference in infection intensities between species was not statistically significant (U = 1020.0, *p* = 0.188), although the golden jackals showed a tendency toward higher larval burdens, driven by several individuals exhibiting very heavy infections, including a juvenile (Figure 4).

### 3.2. Seroconversion Dynamics and Rabies Virus-Neutralising Antibodies (RVNA) Profiles

**Figure 5:**
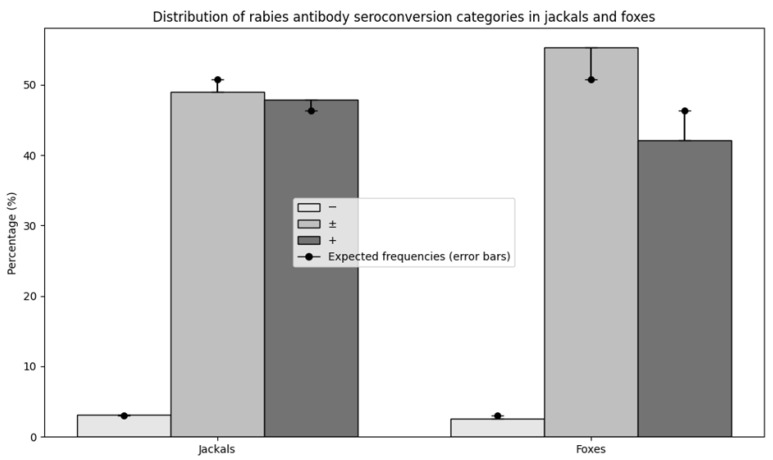
The distribution of rabies antibody seroconversion categories (undetectable −, insufficient ±, and sufficient +) in jackals and foxes; bars—observed frequencies; bidirectional error bars—deviations from expected frequencies under the chi-squared independence model.

In golden jackals, 3.1% (3/96, two males and one juvenile) of samples showed undetectable seroconversion levels (<0.125 IU/mL), 49.0% (47/96) showed insufficient seroconversion (0.125–0.5 IU/mL), and 47.9% (46/96) showed sufficient seroconversion (0.5–4.0 IU/mL). In red foxes, 2.6% (1/38, one juvenile), 55.3% (21/38), and 42.1% (16/38) of samples fell into the undetectable, insufficient, and sufficient seroconversion categories, respectively. There was no significant difference between jackals and foxes (*p* = 0.815) (Figure 5).

When RVNA titers were collapsed into non-protective (<0.5 IU/mL) and protective (≥0.5 IU/mL) categories, no differences between biological categories were observed in either species. In foxes, seroconversion status did not differ among males, females, and juveniles (*p* = 0.350). Similarly, jackals showed no age-related variation in protective responses (χ^2^ = 2.36, *p* = 0.307).

**Figure 6:**
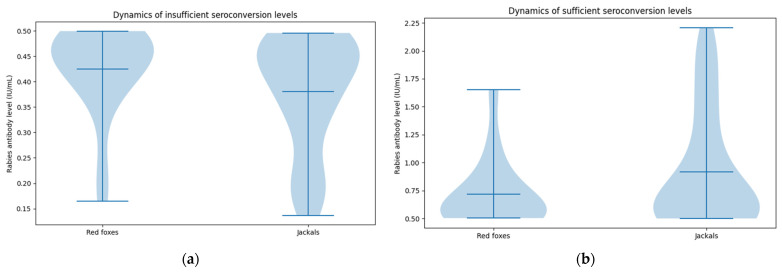
Violin plot illustrating: (**a**) insufficient RVNA titers (below the 0.5 IU/mL protection); Foxes display a narrow distribution, with most individuals clustering close to the upper limit of the insufficient range (0.45–0.50 IU/mL), whereas jackals exhibit a wider spread of values, with more individuals showing low antibody levels (<0.25 IU/mL); (**b**) protective RVNA titers (≥0.5 IU/mL); Fox distributions are moderately tight, with most individuals falling within 0.50–0.80 IU/mL and a few higher responders exceeding 1.0 IU/mL, while jackals show broad variability, a wider and higher range of antibody values and several individuals with exceptionally high RVNA titers.

Red foxes showed a relatively narrow distribution of insufficient antibody titers, ranging from 0.16 to 0.49 IU/mL, with most values clustered between 0.40 and 0.50 IU/mL. The mean titer was 0.42 ± 0.09 IU/mL, and the median was 0.45 IU/mL. In contrast, golden jackals displayed a broader and more heterogeneous distribution, from 0.13 to 0.49 IU/mL. While many values clustered near the upper limit of insufficient seroconversion (similar to foxes), a pronounced tail of individuals with low antibody titers (<0.25 IU/mL) indicated that some jackals displayed minimal antibody responses. The mean antibody titer 0.38 ± 0.11 IU/mL was lower than that of foxes, and the violin plot reveals a bimodal tendency, reflecting divergent response levels within this group (Figure 6a).

Among individuals with sufficient seroconversion levels, red foxes displayed antibody titers ranging from 0.50 to 1.65 IU/mL, with a mean of 0.72 ± 0.31 IU/mL and a median of 0.56 IU/mL. Although several foxes exhibited high titers (>1.0 IU/mL), most remained within a moderate range (0.50–0.80 IU/mL). Golden jackals exhibited a wider and higher range of titers, extending from 0.50 to 2.21 IU/mL, with a mean of 0.92 ± 0.51 IU/mL. While the median titer (0.60 IU/mL) was comparable to that of foxes, the distribution showed substantially greater spread and multiple high values, indicating very strong antibody production in some individuals. The violin plot reveals a sharp density peak around the protective threshold, followed by a long tail of high values (Figure 6b).

When comparing antibody titers across seroconversion classes, there was a clear separation between the insufficient and sufficient groups. Individuals in the sufficient seroconversion category showed significantly higher antibody levels than individuals grouped into the insufficient seroconversion category, in both red foxes (U = 336, *p* = 2.8 × 10^−7^) and golden jackals (U = 2162, *p* = 1.0 × 10^−16^). When comparing species, no significant differences were detected in antibody levels between foxes and jackals within either the insufficient (U = 369.5, *p* = 0.101) or sufficient seroconversion category (U = 300.0, *p* = 0.278).

## 4. Discussion

### 4.1. Prevalence and Ecological Drivers of Trichinella spp. in Wild Canids

The prevalence patterns of *Trichinella* in the two carnivore species indicate potential differences in their contributions to the parasite’s circulation. Although jackals showed a relatively high infection rate, red foxes exhibited a significantly higher prevalence. Similar observations, where foxes display comparatively higher infection levels, have been reported in other European ecosystems [18,24]. Several ecological investigations demonstrated that foxes show consistently high infection rates in northern and central European ecosystems, largely attributed to their frequent scavenging of carrion and their broad dietary niche, which increases the probability of encountering infected tissue [33,34]. Similar trends were reported by Shimalov and Shimalov (2003) [35] in Belarus, where foxes displayed a significantly higher helminth burden, including *Trichinella* spp., than other mesocarnivores, reinforcing the notion that their foraging behaviour elevates exposure risk. Large-scale epidemiological syntheses, such as those by Pozio and Murrell (2006) [24], highlight foxes as key sylvatic reservoirs, playing an important role in maintaining transmission cycles in comparison to species with more selective or less scavenging-oriented diets. More recently, Gherman et al. (2022) [18] documented substantial *Trichinella* spp. circulation in Romanian wildlife, confirming that red foxes consistently represent one of the most heavily infected carnivore hosts in the region.

Several earlier studies from Southeastern Europe report *Trichinella* spp. infection patterns that contrast with the trend observed in our study group. In Serbia, Dmitric et al. (2017) [36] documented a markedly higher infection rate in jackals (approximately 61%), but similar to our prevalence, compared to foxes (around 35%), suggesting that jackals can serve as major reservoirs in certain ecological contexts. This pattern aligns with results from Ćirović et al. 2015 [37], who conducted a large-scale survey on 738 golden jackals and found an overall prevalence of roughly 16.5%, highlighting substantial spatial heterogeneity in infection pressure even within the same region. Additional evidence from Croatia indicates similarly variable dynamics; Balić et al. (2025) [38] reported moderate *Trichinella* prevalence in jackals (~25%) and comparatively lower levels in foxes across some localities. Franssen et al. (2014) [39] documented exceptionally low prevalence in Dutch red foxes (~0.27%), representing the opposite end of the epidemiological spectrum and illustrating how low-endemic regions can drastically reduce infection probabilities across carnivore hosts. Collectively, these findings underscore that the relative roles of jackals and foxes in sustaining the *Trichinella* cycle vary considerably across Europe, shaped by local ecological and environmental conditions.

When compared with previous data from the same geographic region, the markedly higher fox prevalence observed in our study is strikingly elevated relative to the 21.5% prevalence reported by Imre et al. (2015) [22] in the same Arad and Timiș counties in western Romania. In that earlier investigation, infections were dominated by *T. britovi*, and foxes were characterised primarily as reservoirs for the sylvatic cycle, with only a minor epidemiological role in the domestic cycle due to the near absence of *T. spiralis*, the most pathogenic species of the genus. The considerably higher prevalence detected in our fox population may indicate intensified *Trichinella* circulation in these counties over the past decade, potentially driven by ecological changes such as increased fox densities, shifts in scavenging opportunities, or altered interactions with other wildlife reservoirs [5,6,7,8]. However, an important limitation of our study is that we did not perform molecular identification of the *Trichinella* species circulating in our foxes and jackals. As a result, while our prevalence estimates clearly document substantial exposure, we cannot distinguish the relative contributions of *T. britovi* versus *T. spiralis* or assess the extent to which each species participates in the sylvatic versus domestic cycles. Despite this constraint, both our findings and those of Imre et al. (2015) [22] converge on the conclusion that *V. vulpes* remain key contributors to the maintenance of the sylvatic cycle in this region.

Broader ecological and epidemiological studies provide important context for understanding why carnivore species may contribute differently to *Trichinella* spp. transmission. Research on the rapid range expansion of the golden jackal across Europe illustrates how shifting distribution patterns expose this species to new habitats, prey communities, and scavenging opportunities that can either enhance or limit parasite acquisition [40]. Complementary global reviews of *Trichinella* epidemiology emphasize that host involvement in parasite maintenance is shaped by factors such as host density, prey availability, carrion consumption, and sympatric interactions with other wildlife reservoirs [41]. Within this ecological framework, species-specific feeding strategies become key determinants of transmission dynamics. Red foxes exploit carrion more frequently and interact more often with infected carcasses or intermediate hosts [35], potentially elevating their exposure to *Trichinella* larvae. In contrast, jackals, although opportunistic, tend to rely more heavily on live small or even larger prey and vegetal food sources, thereby reducing their likelihood of encountering infected sylvatic reservoirs [42,43].

The significantly higher *Trichinella* prevalence observed in male golden jackals compared with females and juveniles in our study is not consistently supported by the broader literature on the golden jackal. In the largest available jackal-focused dataset to date, Ćirović et al. (2015) [37] examined 738 golden jackals in Serbia and found no difference in prevalence between males and females (16.2% vs. 16.9%), suggesting that sex-related exposure may be weak or context-dependent at broader spatial and temporal scales. Age-related patterns observed in our dataset, with juveniles showing lower infection rates than adults, are consistent with longstanding evidence that *Trichinella* prevalence increases with age, as older individuals accumulate larvae through repeated scavenging events [44,45]. In contrast, studies on other carnivore species—such as wolverines, lynxes, red foxes, and bears—frequently report higher male prevalence, a trend attributed to larger male home ranges, greater scavenging and long-distance foraging behaviour, and increased intrasexual aggression that elevates contact with infected carcasses [44,46]. These behavioural drivers may explain why slightly male-biased tendencies are occasionally observed in regional jackal datasets, even when they do not reach statistical significance. Regardless of sex-specific patterns, golden jackals are increasingly recognized as important reservoirs of *T. spiralis* and *T. britovi* across Europe and western Asia, particularly in landscapes where scavenging of domestic pig carcasses or wild ungulate remains is common [37]. Taken together, the available evidence suggests that while male-biased prevalence is well documented in other carnivore species, the strong sex asymmetry in jackals observed in our sample likely reflects local ecological conditions (e.g., carcass availability, scavenging opportunities, land-use context) and/or sampling structure (notably our comparatively small female sample size), rather than a generalizable biological, sex-linked pattern.

The uniformly high *Trichinella* prevalence observed among male, female, and juvenile red foxes in our study, coupled with the close agreement between expected and observed frequencies, indicates that exposure is broadly homogeneous across biological categories. Studies on *Trichinella* prevalence in European red foxes indicate that while infection rates can be high in certain regions, in terms of sex, the infection frequency is often similar between males and females [36,47]. However, the distribution is not always uniform across all age groups, with older foxes often showing higher prevalence than juveniles [34,48]. This discrepancy is typically attributed to cumulative exposure: adults have had more opportunities to encounter infected carcasses or prey over their lifetime, increasing their likelihood of acquiring the parasite. Additionally, older foxes may display more pronounced scavenging behaviour or broader home ranges, further elevating exposure risk. Juveniles, in contrast, may rely more on parental provisioning or limit their movements to natal territories, reducing their immediate contact with infected sources during their early months [34,49]. Against this background, the relatively uniform prevalence across age groups in our dataset may indicate region-specific ecological dynamics. For example, if *Trichinella* infection pressure in the local environment is particularly high, even young foxes may quickly encounter infected prey or carrion. Alternatively, early independent foraging behaviours or high local carcass availability could equalise exposure opportunities between juveniles and adults [21].

### 4.2. Intensity of Trichinella Infections and Larval Burdens

The present comparison of *Trichinella* larval burdens in golden jackals and red foxes showed similar infection intensities, with no statistically significant interspecific difference, although jackals tended to display higher mean and maximum LPG values. This pattern is consistent with previous studies indicating that jackals often act as highly competent hosts within both the domestic and sylvatic cycles of *Trichinella* spp. across Europe [37,38]. In our dataset, jackals showed a wide range of infection intensities (0.2–78.8 LPG), including occasional extremely heavy infections, a phenomenon also documented in Serbia, Hungary, and Croatia, where individual jackals frequently harbour high larval loads [37,38,50]. Juvenile jackals in our study exhibited generally low burdens, with only one notable outlier. Similar age-related patterns have been observed in other carnivores, where cumulative exposure leads to increasing larval densities with age [44,51].

Red foxes showed similar variable burdens (0.4–55.4 LPG), with several moderately to heavily infected adults, which aligns with prior findings demonstrating that foxes remain key reservoirs of *Trichinella* spp. throughout Europe [36,52]. Although foxes are traditionally considered the principal sylvatic reservoir, recent work emphasizes that jackals may exceed foxes in epidemiological importance in some regions due to their expanding range and scavenging habits [38].

The absence of significant sex-based differences in either species corresponds to the majority of European studies reporting similar prevalence and intensity between males and females [36,37], though small sample sizes, particularly among adult female jackals, limit interpretation. Importantly, several *Trichinella* taxa circulate in the region, including *T. spiralis*, *T. britovi*, *T. nativa*, and occasionally *T. pseudospiralis*, with the latter recently reported from a Romanian jackal [23]. Overall, our findings support the growing body of evidence that both golden jackals and red foxes function as major sylvatic hosts sustaining *Trichinella* transmission. Their similar larval burdens and overlapping ecological niches highlight the need for continued surveillance in both species, especially given the ongoing expansion of jackal populations across Europe and their demonstrated involvement in the domestic cycle [53].

### 4.3. Rabies Seroconversion Dynamics

Rabies vaccination in wildlife in Romania focuses primarily on red foxes, the main reservoir for the rabies virus in the region [25]. Because Romania borders non-EU countries where rabies remains endemic, the country conducts regular, EU-co-financed, and scientifically monitored oral rabies vaccination (ORV) campaigns [54]. Since 2012, biannual ORV campaigns, implemented each spring and autumn, have covered more than 237,000 km^2^ nationwide, using vaccine baits distributed mostly by air with targeted manual distribution in areas inaccessible to aircraft [25].

The baits contain a live attenuated vaccine strain (SAD Bern or SAD B19), which induces protective immunity following ingestion [54]. Although foxes are the primary target species, bait competition is common; studies show that wild boars frequently consume ORV baits, and high post-campaign seroprevalence has been reported in this species [25]. ORV effectiveness is monitored through sampling of hunted foxes (approximately four foxes/100 km^2^) beginning 45 days after bait distribution, assessing rabies virus-neutralising antibodies via ELISA and evaluating tetracycline deposition as evidence of bait consumption [25].

A central finding of this study is that more than half of all tested individuals (57.9% of the red foxes and 52.0% of the golden jackals) exhibited RVNA titers below the protective threshold of 0.5 IU/mL. This substantial proportion of animals with insufficient antibody titers highlights the complexity of rabies immunity in free-ranging carnivores and underscores the need to interpret serological data cautiously [55]. The distribution of antibody titers further emphasises species-specific patterns. Red foxes exhibited a relatively narrow distribution of insufficient RVNA titers, with most values clustering near the protective threshold (0.40–0.50 IU/mL) and moderate variation among the sufficient seroconversion group (0.50–1.65 IU/mL). This pattern is broadly consistent with seroconversion profiles documented in European ORV monitoring programs, where fox populations typically achieve high bait uptake and substantial antibody responses following systematic vaccination [25,54]. In contrast, golden jackals showed a wider spread of insufficient titers and a notably greater range of high titers among individuals in the sufficient seroconversion group, suggesting both heterogeneity in vaccine uptake and a potential for strong individual immune responses. Similar variability in immune response has been reported in jackal populations targeted with oral vaccines in other regions, including South Africa, where protective antibody responses were observed in a subset of free-ranging jackals [28]. However, both species demonstrated clear immunological separation between insufficient and sufficient seroconversion classes, with sufficient individuals producing significantly higher antibody titers. Despite different distribution patterns, no significant differences were found between species within either seroconversion class, indicating similar central tendencies in immune response. Importantly, the present study was not designed to evaluate the effectiveness of Romania’s oral rabies vaccination (ORV) campaigns, and antibody levels reflect the immune landscape of the sampled animals at a single point in time, which might be influenced by factors such as bait competition, ecological behaviour, natural exposure history and methodological factors, rather than a direct measure of ORV performance.

The high proportion of sub-protective titers remains relevant in the broader epidemiological context of Romania, where rabies control has historically depended on large-scale ORV programs. Past reports from the European Commission highlight that consistent ORV implementation was essential to reduce rabies incidence in wildlife, with some counties (e.g., Bistrița-Năsăud) reporting several consecutive years without rabies-positive foxes during uninterrupted campaigns [19]. On the other hand, the finding that half of the individuals tested failed to reach protective antibody levels is particularly concerning in the context of recent rabies dynamics in Romania. Despite the overall success of ORV in reducing rabies incidence, historical interruptions to vaccination efforts (2018, 2021, and 2022) have coincided with resurgent rabies activity, including 28 confirmed animal outbreaks and a fatal human case reported in July 2025 [26]. These episodes highlight the fragility of rabies control in the absence of sustained campaign continuity and the importance of maintaining high levels of protective immunity in wildlife reservoirs, as suboptimal seroconversion may reduce population immunity below the critical threshold necessary to interrupt transmission chains, enabling the virus to persist or re-emerge. Moreover, wild boars, which frequently compete with foxes and jackals for baits [25,56], may further reduce vaccine availability for target species.

In this context, the golden jackal, in particular, deserves special attention. Their populations have expanded markedly in Romania over the last decade, facilitated by landscape changes, mild winters, and flexible foraging strategies [57,58]. Recent management actions in the Danube Delta, where jackals were extracted following public safety concerns [59], illustrate the increasing interactions between jackals, human communities, and domestic animals. This expanding distribution not only elevates their ecological significance but also increases their relevance within rabies surveillance. Kemenszky et al. (2020) [27] emphasize that golden jackals require increased attention in rabies surveillance and control due to their expanding role as mesocarnivores capable of bridging ecological zones. Furthermore, their study highlights that ORV strategies originally designed for red foxes may not fully account for jackal-specific ecology, and that explicitly integrating jackals into surveillance and vaccination planning is essential to prevent gaps in population immunity and safeguard long-term rabies control efforts across southeastern Europe [27]. The variability observed in jackal antibody titers in our study, ranging from very low to exceptionally high values, along with the comparatively uniform response in foxes, aligns with the long-standing focus of Romanian ORV campaigns on the last ones and with previous observations that jackals may show inconsistent bait uptake but are capable of strong immune responses when exposure occurs [27,28].

The interpretation of antibody titers requires consideration of broader rabies ecology, including the possibility of naturally acquired RVNAs in unvaccinated individuals. Gold et al. (2020) [55] discuss how rabies-specific antibodies can be detected in healthy, unvaccinated domestic animals and wildlife, potentially indicating nonlethal exposure to rabies virus or other lyssaviruses or limitations of serologic assays. They highlight that variation in test methods and cutoffs complicates the interpretation of seroprevalence data, and that assay specificity and sensitivity must be carefully considered when inferring immunity or exposure from antibody titers [55]. In our study, ELISA and neutralisation assays were interpreted relative to the protective cutoff of 0.5 IU/mL; however, variation in detection thresholds and cross-reactivity with other lyssaviruses [17] could influence measured titers and must be acknowledged when comparing across species or studies.

### 4.4. Study Limitations

The present findings should be interpreted in light of several shared methodological constraints. Although seven hunting grounds were included, all were located in western Romania; therefore, the reported *Trichinella* prevalence and rabies virus-neutralising antibody (RVNA) profiles reflect regional ecological and epidemiological conditions. Local factors such as carnivore density, scavenging opportunities, carcass management practices, proximity to domestic pig holdings, and the intensity and continuity of ORV campaigns may influence both parasite transmission pressure and rabies exposure patterns. Consequently, the levels of *Trichinella* circulation and the proportion of sub-protective RVNA titers observed in this study cannot be directly extrapolated to other Romanian regions, particularly northeastern or southern areas, where habitat structure, wildlife community composition, cross-border rabies dynamics, and vaccination coverage may differ substantially [4,8,18,25]. Sampling was conducted over a six-month period, providing only a temporal snapshot, without assessment of seasonal variation or long-term trends. Some demographic subgroups, particularly adult female jackals and juvenile foxes, were represented by small sample sizes, limiting statistical power in sex- and age-specific analyses. Animals were obtained through routine legal hunting rather than systematic wildlife sampling, which may introduce selection bias toward certain age classes or more visible individuals. Also, key ecological drivers such as carcass availability, prey density, habitat fragmentation, and anthropogenic food resources were not quantitatively measured, meaning that associations between infection patterns and environmental determinants remain inferential rather than directly demonstrated.

An additional limitation concerns the characterization of *Trichinella* infections. No molecular typing of recovered larvae was performed; therefore, the relative contribution of *T. britovi*, *T. spiralis*, *T. pseudospiralis*, or other circulating taxa could not be determined. As a result, it was not possible to distinguish more precisely between strictly sylvatic transmission and potential links to the domestic cycle. Furthermore, although diaphragmatic pillars and intercostal muscles represent standard and recommended sampling sites for routine surveillance, larval distribution in wild carnivores is not uniform across muscle groups. Previous studies indicate that higher larval burdens are frequently detected in the tongue and foreleg muscles, which are considered predilection sites in several carnivore species [36,52,60]. Restricting sampling to a single anatomical region per individual may therefore have led to underestimation of total parasite burden, particularly in lightly infected animals.

Finally, several limitations should be noted for the assessment of RVNA profiles in our wild canid population. First, serological data provide a snapshot of antibody presence but do not directly measure long-term protection or actual exposure risk (e.g., absence of confirmatory data on individual bait ingestion). As Gold et al. (2020) emphasize, false positives and variability in test performance may result in over- or underestimation of true immune status, particularly when comparing distinct species [55]. Second, the sample sizes in some demographic groups were relatively small, limiting the power to detect subtle differences. Third, the reliance on a single protective cutoff (0.5 IU/mL) may not capture species-specific thresholds of protective immunity, especially in wildlife species for which this value has not been fully validated.

## 5. Conclusions

This study demonstrates intense sylvatic circulation of *Trichinella* spp. in western Romania, with remarkably high prevalence in both red foxes (78.9%) and golden jackals (60.4%). With statistically significant interspecific differences, red foxes reinforce their role as primary sylvatic reservoirs. Concurrently, the substantial infection levels and expanding distribution of the golden jackals highlight their growing epidemiological relevance as competent maintenance hosts capable of sustaining and potentially amplifying transmission.

From a rabies perspective, more than half of the sampled individuals displayed antibody titers below the protective threshold (0.5 IU/mL), raising concerns regarding population-level immunity. While red foxes remain the principal target of oral rabies vaccination (ORV) campaigns, the ecological expansion and synanthropic behaviour of golden jackals underscore the need for their systematic inclusion in future disease surveillance programs. Ultimately, these findings reinforce the value of comprehensive, multi-pathogen surveillance in ecologically overlapping carnivore populations in order to guide evidence-based wildlife management and mitigate zoonotic risks at the wildlife–domestic–human interface.

## Data Availability

Data is contained within the article.

## Abbreviations

The following abbreviations are used in this manuscript:

RVNA	Rabies Virus Neutralising Antibody
ORV	Oral Rabies Vaccination
LPG	Larvae Per Gram
